# Bibliometric analysis of photodynamic therapy and immune response from 1989–2023

**DOI:** 10.3389/fphar.2024.1299253

**Published:** 2024-01-15

**Authors:** Wanting Fan, Jianming Tang, Su Tang, Zhengshen Lin, Mohan Li, Zheng Zhang, Donglei Wu

**Affiliations:** ^1^ Department of Stomatology, Shenzhen People’s Hospital, Shenzhen, China; ^2^ Department of Stomatology, The People’s Hospital of Baoan Shenzhen, The Second Affiliated Hospital of Shenzhen University, Shenzhen, China

**Keywords:** bibliometric analysis, photodynamic therapy, antitumor immunity, immunogenic cell death, visualization

## Abstract

**Objective:** Photodynamic therapy (PDT) is a minimally invasive treatment approach for precancerous and cancerous lesions, known for its ability to activate the host immune response. This study conducted a bibliometric analysis to identify the research trends and hotspots related to the immune response in PDT.

**Methods:** We analyzed articles and reviews published from 1989 to 2023, retrieved from the Web of Science database. Using Citespace and VOSviewer, we visualized the distribution patterns of these studies in time and space.

**Results:** The analysis revealed a substantial increase in the number of publications on PDT-related immune response since 1989. A total of 1,688 articles from 1,701 institutions were included in this analysis. Among thei nstitutions, the Chinese Academy of Sciences demonstrated exceptional productivity and a willingness to collaborate with others. Additionally, 8,567 authors contributed to the field, with Mladen Korbelik, Michael R. Hamblin, and Wei R. Chen being the most prolific contributors. The current research focus revolves around novel strategies to enhance antitumor immunity in PDT, including PDT-based dendritic cell vaccines, combination therapies with immune checkpoint inhibitors (ICIs), and the use of nanoparticles for photosensitizer delivery. Furthermore, genes such as CD8A, TNF, CD4, IFNG, CD274, IL6, IL10, CALR, HMGB1, and CTLA4 have been evaluated in the context of PDT-related immunity.

**Conclusion:** PDT not only achieves tumor ablation but also stimulates the immune response, bolstering antitumor immunity. This study highlights the emerging hotspots in PDT-related immune response research and provides valuable insights for future investigations aimed at further enhancing antitumor immunity.

## 1 Introduction

Photodynamic therapy (PDT) is a highly selective, minimally invasive, and low-toxicity treatment for precancerous lesions and solid tumors (Falk-Mahapatra and Gollnick, 2020). Extensive preclinical and clinical research has confirmed the effectiveness of PDT in cancer therapy, it can treat various benign and malignant tumors of digestive system, respiratory system, urinary system, gynecology, ENT department, dermatology and neurosurgery, including cutaneous squamous cell carcinoma, basal cell carcinoma, lung cancer, and glioma (Agostinis et al., 2011). Additionally, PDT serves as a palliative therapy for advanced and inoperable malignancies, significantly improving patients’ quality of life (Moghissi et al., 2000; Zou et al., 2020). PDT involves three key components: the photosensitizer (PS), light, and molecular oxygen. The PS is selectively absorbed by neoplasms and precancerous lesions, leading to the production of reactive oxygen species (ROS) upon irradiation with light of specific wavelengths, thereby playing a crucial role in cancer elimination. With the emergence of immunotherapy and targeted therapy for cancer and precancerous conditions, PDT has garnered attention from clinical researchers. An ever-growing body of research suggests that PDT induces cancer ablation by triggering immune responses (Tan et al., 2022; Lu et al., 2023). Various inflammatory mediators and immune cells are implicated in PDT-induced immunogenic cell death (ICD) (Alzeibak et al., 2021). In recent decades, the number of studies investigating the relationship between PDT and immunity has rapidly increased. However, the temporal and spatial distribution characteristics of these studies have seldom been discussed. Consequently, the objective of this study is to examine the research hotspots and trends in PDT-related immune responses through bibliometric analysis. This research aims to provide readers with a concise and captivating overview of the essential information, analyses, and arguments presented throughout the paper, facilitating their comprehension and retention of its key points.

## 2 Materials and methods

### 2.1 Data source and search strategy

All data for this study was collected from the Web of Science Core Collection (https://clarivate.com/), which is a widely recognized and comprehensive citation database that includes scholarly journals, books, book series, and conference proceedings. The data retrieval was conducted on 1 March 2023. To gather relevant articles, a search formula based on subject terms was employed. The formula consisted of two main components: #1: TS = (“photodynamic” OR “photodynamic therapy” OR “photodynamic therapies”) and #2: TS = (“immunity” OR “immune response” OR “immune regulation”). The final dataset was obtained by combining the results of #1 and #2 using the logical operator “AND”. The search was limited to articles published in the English language, and the time span was restricted from 1989 to 2023 to encompass the first related article published in 1989. To ensure the inclusion of substantial research, the literature type was limited to articles and reviews, while conference abstracts, conference proceedings, and letters were excluded from the analysis. For further bibliometric analysis, information such as authors, institutions, regions, countries, and references of each article were exported to facilitate visualization and comprehensive examination.

### 2.2 Data analysis

To analyze the data, several software tools were utilized. The VOS viewer 1.6.18 (https://www.vosviewer.com/) was employed for co-occurrence analysis of countries, institutions, and authors. Additionally, the CiteSpace 6.16 (https://citespace.podia.com/) software was utilized for co-occurrence network analysis, timeline view, and burst word analysis. Cocitation analysis and cluster analysis were conducted to examine the intellectual foundation of PDT-related immune response research. Cocitation analysis measures the frequency with which two references are cited together (Small, 1973). To identify significant clustering structures, modularity (Q value >0.3) and weighted mean silhouette (S value >0.5) were considered. Keywords co-occurrence network analysis and timeline view were utilized to identify emerging topics and potential future directions in the field.

For the visualization of gene co-occurrence analysis and disease co-occurrence analysis related to PDT-induced immune regulation, VOSviewer was employed. These analyses aim to provide insights into the relationships and patterns within the research literature.

## 3 Results

### 3.1 Chronological and spatial distribution in PDT related immune response

**FIGURE 1:**
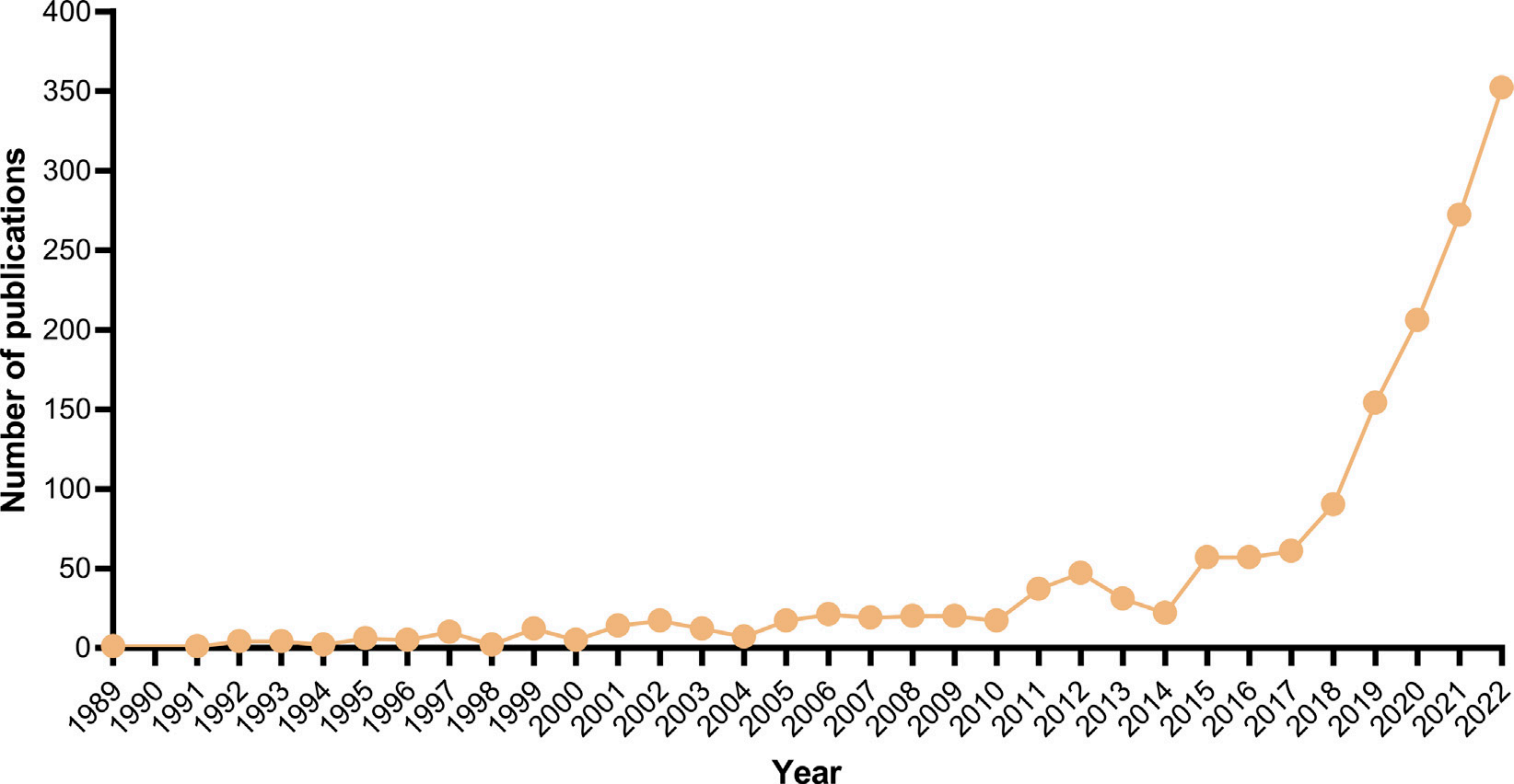
Number of published articles on PDT related immune response in the period of 1989–2022.

A comprehensive total of 1,688 articles were included in this research, covering the time period from 1989 to 2023. The first article related to PDT and immunity was published in 1989 (Nseyo et al., 1989). As depicted in Figure 1, the number of articles has shown a consistent upward trend since 1989. Particularly, there has been a significant increase in the number of publications since 2018, with over 50 articles being published annually after 2014.

### 3.2 Inter-institutional collaboration network

**FIGURE 2:**
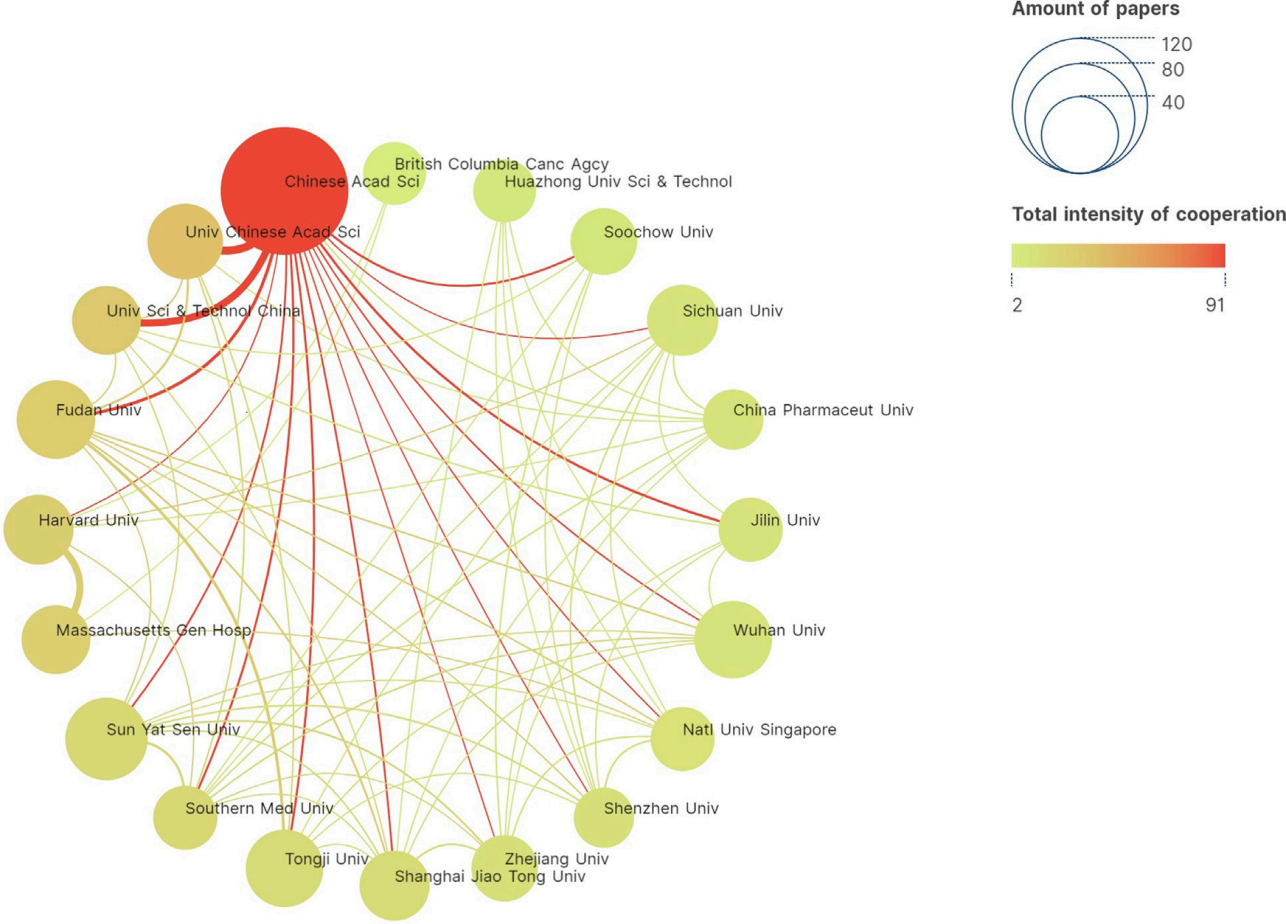
Inter-institutional collaboration network diagram conducted by VOSviewer. Top 20 productive institutions are shown. The node represents institution, while the line represents collaboration. The size of node represents the number of publications, and the thickness of line represents the strength of collaboration. The color of node represents the intensity of cooperation.

The analysis of inter-institutional collaboration in the field of PDT-related immune regulation revealed important insights into the research landscape. A total of 1701 institutions have contributed to the 1,688 publications on this topic. Among these institutions, the Chinese Academy of Sciences emerges as the most productive, with an impressive publication count of 103 related articles. Following closely is Sun Yat-Sen University, which ranks second in terms of publication output. Furthermore, the analysis indicates a close level of inter-institutional collaboration within the field of PDT-related immune response. Institutions exhibit a strong willingness to collaborate, fostering a network of scientific cooperation. Notably, the Chinese Academy of Sciences demonstrates a significant inclination to collaborate with other institutions, with a particularly strong partnership observed with the University of Chinese Academy of Sciences (Figure 2).

### 3.3 Cooperative relationship between authors

**FIGURE 3:**
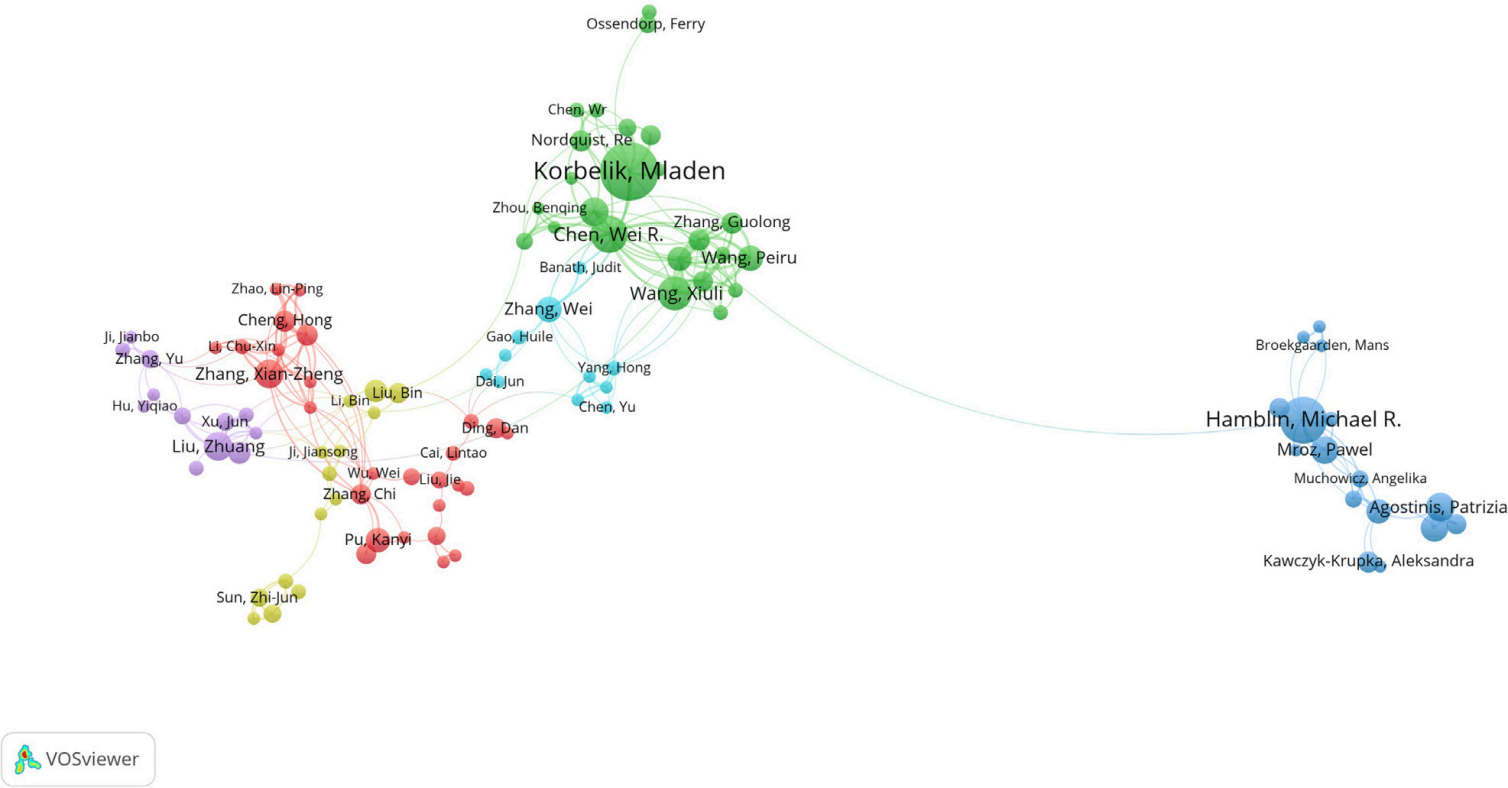
Cooperative relationship between authors conducted by VOSviewer. The node represents author, while the line represents cooperative relationship. The size of node represents the number of publications, and the thickness of line represents the strength of collaboration. The color of node represents the cluster.

In the analysis of the cooperative relationship between authors in the field of PDT-related immune response, a total of 8,567 authors contributed to the 1,688 related articles. Figure 3 highlights the cooperative relationships among 104 authors who have published at least 5 articles. The analysis reveals that Mladen Korbelik has published the largest number of papers, followed by Michael R. Hamblin and Wei R Chen. These authors have made significant contributions to the field. Furthermore, the authors can be grouped into 6 clusters based on cluster detection, indicating close cooperation within each cluster. This suggests that authors within the same cluster have collaborated closely on research projects and publications. Moreover, within the network of authors, Wei R Chen and Feifan Zhou exhibit the most closely cooperative relationship, indicating a strong collaborative partnership between these two authors.

### 3.4 Cited journals with the strongest citation bursts

**FIGURE 4:**
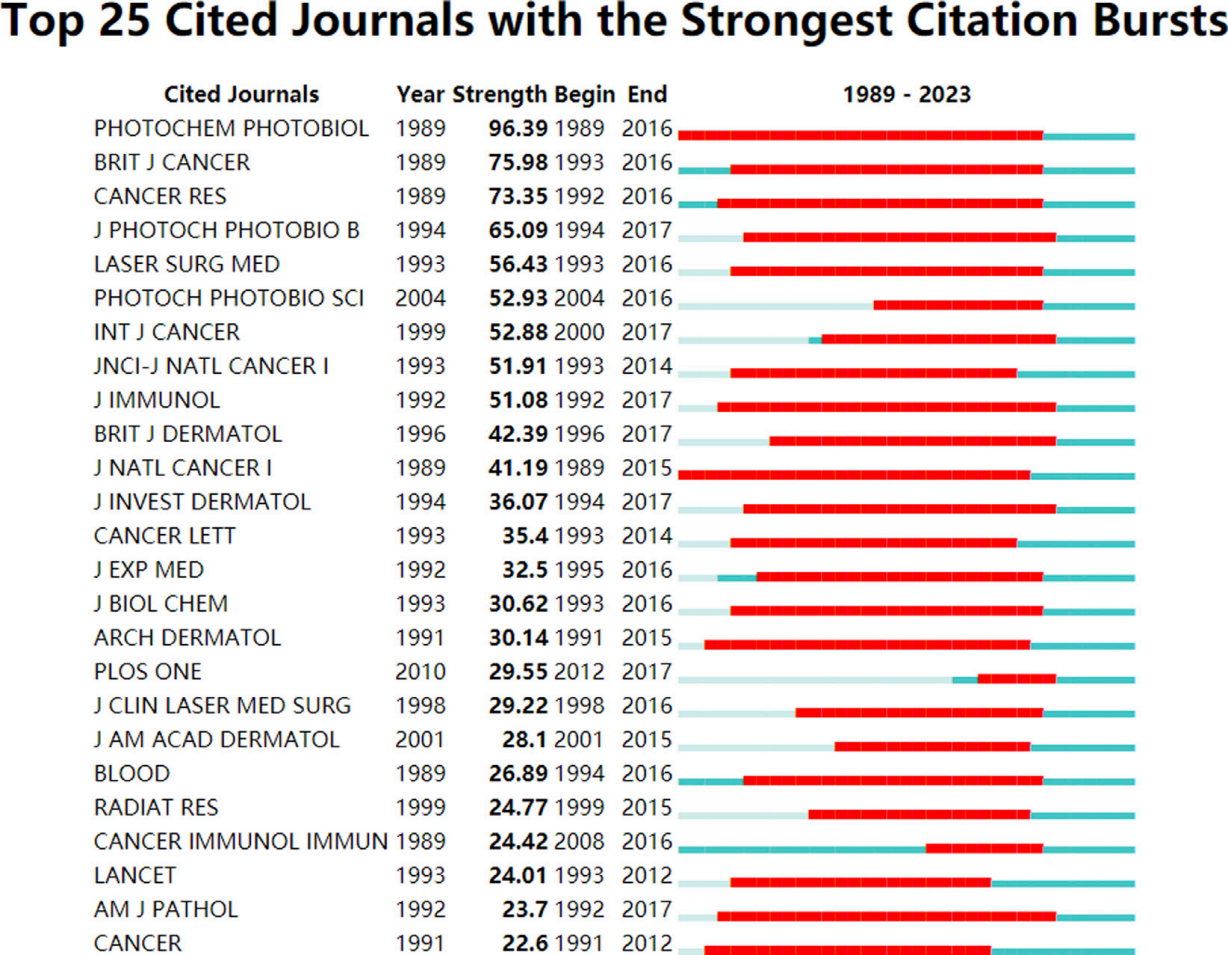
Top 25 cited journals with the strongest citation bursts conducted by CiteSpace. The red zone represents the strongest citation burst at the respective journal during 1989–2023.

To further explore the citation impact in the field, the analysis visualizes the number of cited journals with the strongest citation bursts using CiteSpace. Figure 4 highlights the journal “Photochemistry and Photobiology” with the strongest citation burst from 1989 to 2016, indicating its high impact and influence in the field, with a strength value of 96.39.

### 3.5 Co-citation analysis for references

**FIGURE 5:**
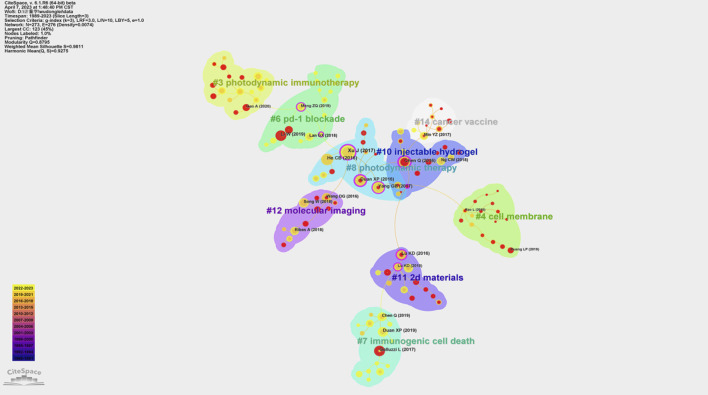
Co-citation analysis conducted on Citespace. The node represents cited article. The size of node represents the number of citations. Purple circle around the node represents reference with centrality >0.1. The line between nodes represents co-cited relationships. Different color blocks mean different clusters.

Figure 5; Table 1 present the results of the co-citation analysis for references in the field of PDT-related immune response. The most highly cited publication, authored by Xu (Xu et al., 2017), which has been cited 176 times. Xu et al. (Xu et al., 2017) introduced a novel PDT strategy that utilizes immune-stimulating upconversion nanoparticles (UCNP) in combination with CTLA-4 checkpoint blockade. This strategy aimed to eliminate colorectal cancer, prevent tumor recurrence, and leverage the immune memory effect. The second most highly cited publication, authored by Yang (Yang et al., 2017), has been cited 118 times. Yang (Yang et al., 2017) developed a tumor microenvironment (TME) responsive nano-platform loaded with the photodynamic agent chlorine e6 (Ce6) and the chemotherapy drug doxorubicin. This platform effectively triggered a series of anti-tumor immune responses and inhibited tumor growth. Additionally, the co-citation analysis revealed the presence of 9 co-citation clusters, as determined by cluster analysis. These clusters represent thematic groupings within the field and include topics such as photodynamic immunotherapy (#3), cell membrane (#4), PD-1 blockade (#6), immunogenic cell death (#7), photodynamic therapy (#8), injectable hydrogel (#10), 2D materials (#11), and molecular imaging (#12).

**TABLE 1:** Top 10 cited references in PDT of immunity.

Cited reference	Cited times	Cluster	Centrality	Year	Source
Xu J (Nseyo et al., 1989)	176	8	0.28	2017	ACS NANO
He CB (Yang et al., 2017)	127	8	0.02	2016	NAT COMMUN
Yang GB (Xu et al., 2017)	118	8	0.1	2017	NAT COMMUN
Chen Q (Deets and Vance, 2021)	117	10	0.23	2016	NAT COMMUN
Duan XP (Dumauthioz et al., 2018)	104	8	0.21	2016	J AM CHEM SOC
Galluzzi L (Pulendran and Davis, 2020)	103	7	0.04	2017	NAT REV IMMUNOL
Duan XP (Donohoe et al., 2019)	102	7	0.05	2019	ANGEW CHEM INT EDIT
Li W (Korbelik et al., 2014)	99	6	0.01	2019	NAT COMMUN
Lu KD (Gollnick et al., 1997)	91	11	0.14	2016	J AM CHEM SOC
Wang DG (Gollnick et al., 2001)	81	12	0.06	2016	NANO LETT

### 3.6 Keywords co-occurrence network analysis

**FIGURE 6:**
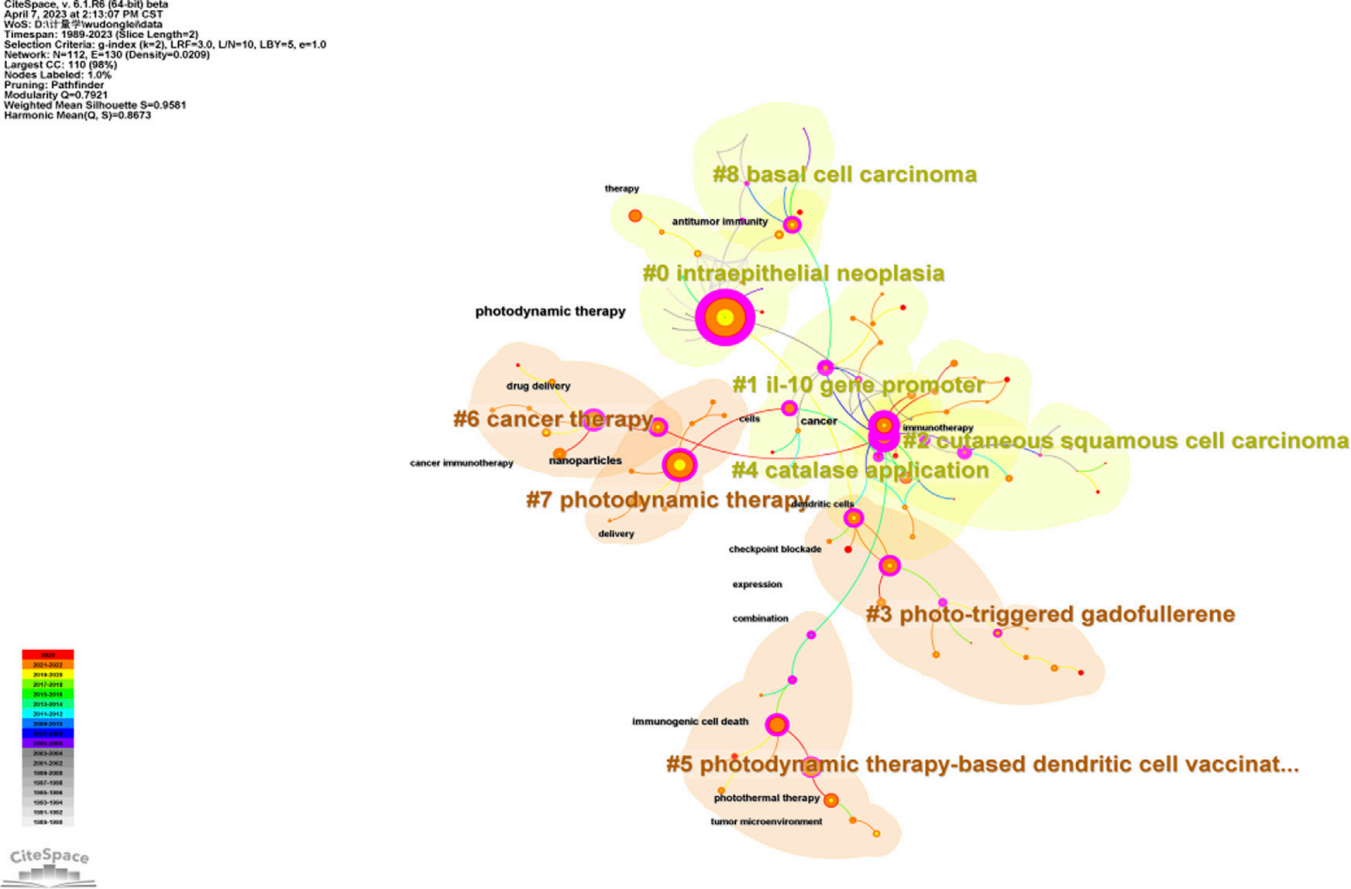
Keywords co-occurrence network analysis constructed on CiteSpace. The circle represents keywords. The overall size of the circle reflects the frequency of keywords. The color of the circle represents the corresponding time.

The keyword co-occurrence analysis identified a total of 113 keywords in the field of PDT-related immune response. Among these keywords, PDT had the highest frequency of occurrence in the cluster of intraepithelial neoplasia, with its first appearance noted in 1992 (Table 2). Furthermore, keywords such as cells and expression were frequently detected in the cluster related to the IL-10 gene promoter. The analysis also revealed the emergence of nano materials and nanoparticles carrying photosensitizers (PS) in PDT for cancer therapy since 2015. This indicates the growing utilization of nanotechnology in enhancing the effectiveness of PDT. The 9 clustered keywords identified in the analysis were as follows: intraepithelial neoplasia (#0), IL-10 gene promoter (#1), cutaneous squamous cell carcinoma (#2), photo-triggered gadofullerene (#3), catalase application (#4), PDT-based dendritic cell (DC) vaccination (#5), cancer therapy (#6), photodynamic therapy (#7), and basal cell carcinoma (#8) (Figure 6). These clustered keywords represent specific areas of focus within the field, including different types of tumors and novel treatment strategies related to PDT and immune response.

**TABLE 2:** Top 10 co-occurrence keywords in PDT related immunity.

Keywords	Frequency	Degree	Centrality	Year
photodynamic therapy	1,100	17	0.93	1992
nanoparticles	306	5	0.24	2015
cancer	269	6	0.77	1999
immunogenic cell death	191	4	0.27	2015
drug delivery	163	5	0.21	2015
antitumor immunity	163	4	0.23	2005
photothermal therapy	154	2	0.1	2017
dendritic cells	143	8	0.95	2005
cancer immunotherapy	134	1	0	2017
delivery	133	2	0.04	2017

**FIGURE 7:**
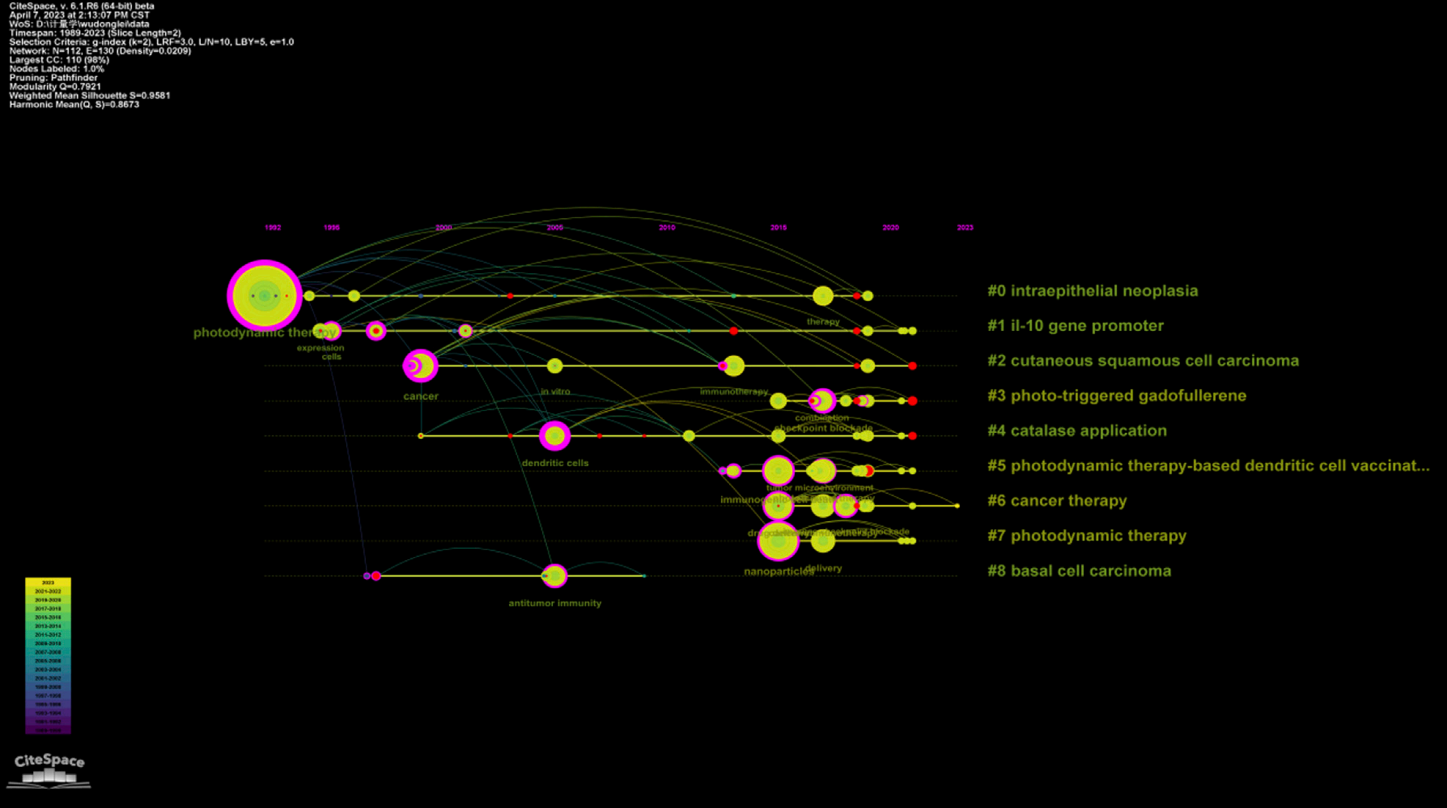
Timeline view for keywords in each cluster by Citespcace. Horizontal axis in the timeline view represents cluster. The position of the node on the horizontal axis represents the time point of the first appearance. The camber line between nodes represents co-cited relationships. The size of node indicates the frequency of occurrence.

The analysis also indicates a significant increase in the number of articles in several clusters since 2015, such as those related to photo-triggered gadofullerene (#3), catalase application (#4), PDT-based dendritic cell (DC) vaccination (#5), cancer therapy (#6), and photodynamic therapy (#7). This suggests a growing interest and research focus on these topics. Furthermore, the indication of studies on PDT-related immunity has expanded from precancerous conditions to cancer as the years progress (Figure 7). This demonstrates the evolving research landscape and the broadening application of PDT in the context of immune response.

#### 3.7 Genes cooccurrence analysis

**FIGURE 8:**
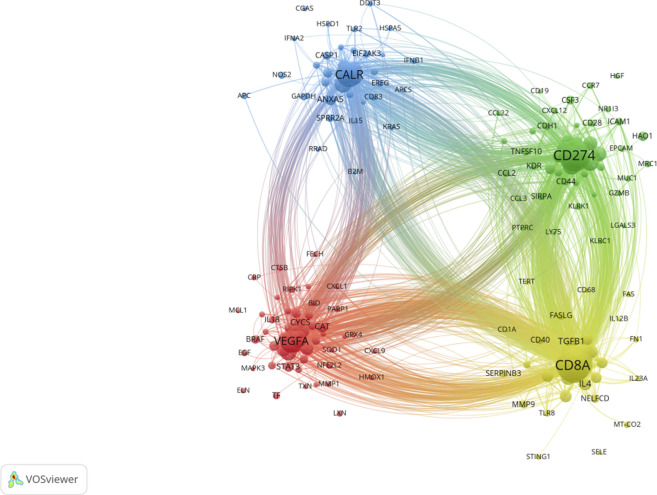
Gene cooccurrence network analysis in PDT related immune regulation. The node represents the gene with frequency of occurrence over than 8. The size of node represents the frequency of occurrence. The degree of line between nodes represents the strength of correlation. Different colors of nodes represent different clusters.

The genes co-occurrence analysis revealed a total of 169 genes that were enriched in the context of PDT-related immune regulation. These genes were divided into 4 distinct clusters. Among these genes, the top 10 genes with high weight were identified as follows: CD8A, TNF, CD4, IFNG, CD274, IL6, IL10, CALR, HMGB1, and CTLA4. The CD8A gene, which is primarily expressed in cytotoxic T lymphocytes, was found to be involved in the yellow cluster. This suggests its significance in the immune response associated with PDT-related immune regulation. CD274, which encodes an immune inhibitory receptor ligand known as PD-L1, was clustered in the green cluster. This highlights its role in modulating immune responses in the context of PDT. VEGFA, a member of the platelet-derived growth factor (PDGF) family, was clustered in the red cluster. This indicates its potential involvement in angiogenesis and vascular-related processes within the scope of PDT-related immune regulation. CALR, which encodes the calreticulin protein, was clustered in the blue cluster, suggesting its relevance to cellular processes and immune responses (Figure 8).

#### 3.8 Diseases cooccurrence analysis

**FIGURE 9:**
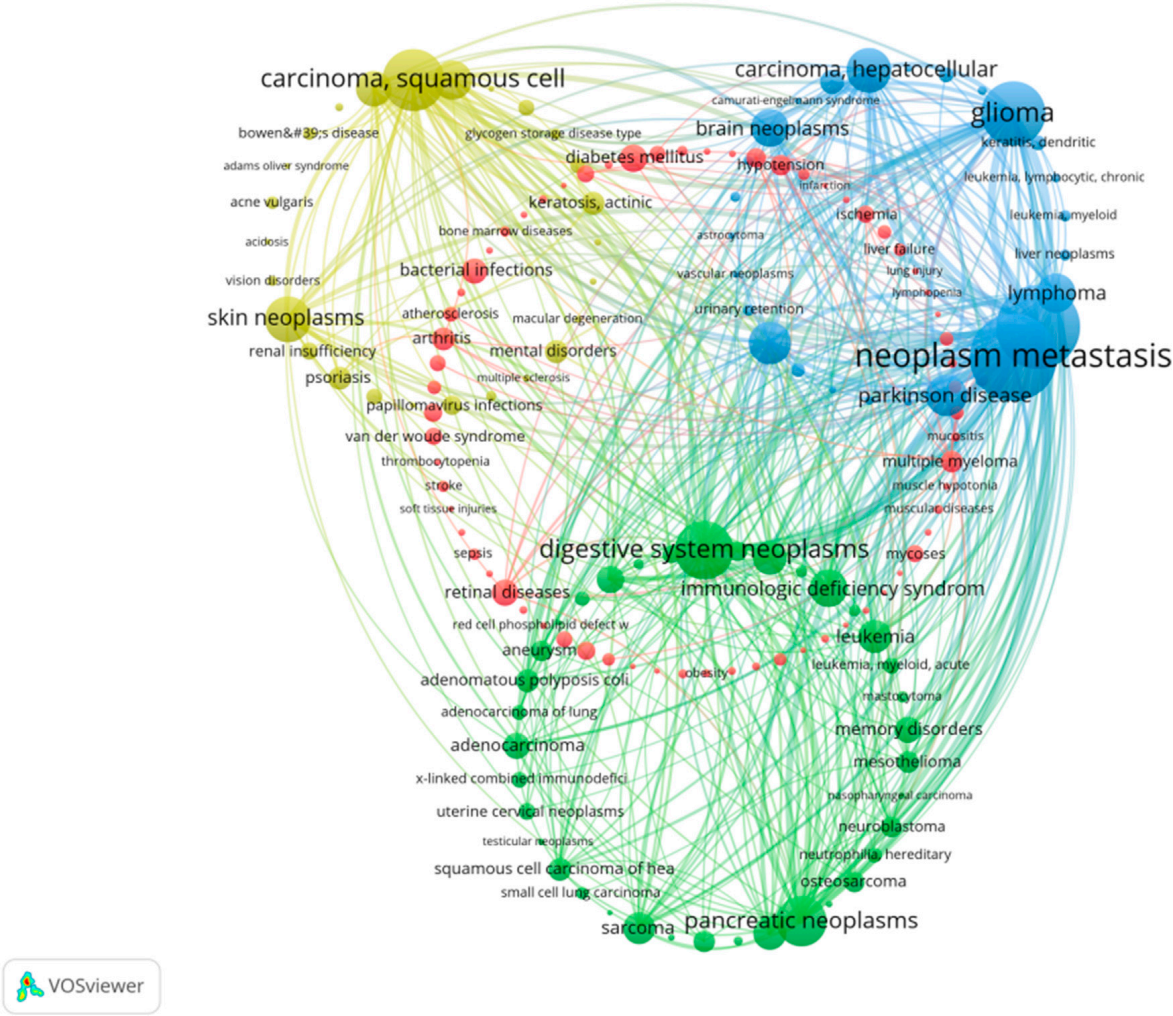
Diseases cooccurrence network analysis in PDT related immune regulation. The node represents the gene with frequency of occurrence over than 8. The size of node represents the frequency of occurrence. The degree of line between nodes represents the strength of correlation. Different colors of nodes represent different clusters.

A total of 156 diseases were discussed in PDT related immune respnse, and they were divided into four distinct clusters. Among these diseases, the top 10 diseases with high weight were identified as follows: melanoma, metastatic neoplasm, glioma, digestive system neoplasms, squamous cell carcinoma, pancreatic neoplasms, hepatocellular carcinoma, urinary bladder neoplasms, lymphoma, and skin neoplasms (Figure 9).

## 4 Discussion

This bibliometric analysis shed light on chronological and spatial distribution in PDT related immune response from 1989 to 2023. The collaborative network signifies a vibrant research community dedicated to advancing knowledge in PDT-related immune response. In addition, collaborative efforts among institutions contribute to the exchange of ideas, expertise, and resources, fostering a fertile ground for scientific progress.

The immune state of the body, especially the strength of cellular immune function, is closely related to the occurrence of tumors (Deets and Vance, 2021). When the immune function of the body is low or suppressed, the incidence of tumors will often increase (Dumauthioz et al., 2018). After the occurrence of tumor, the body may have strong or weak specific and non-specific immune responses, but due to the weak immunogenicity of tumor cells, it is difficult to trigger sufficient anti-tumor immune effects (Pulendran and Davis, 2020). The immune effect induced by PDT is mainly achieved through two ways: (1) Activating the innate immunity of the body. After PDT treatment, acute inflammation will be triggered, neutrophil infiltration will be promoted, and the innate immunity of the body will be activated (Donohoe et al., 2019). (2) Activating the body’s specific immunity and trigger ICD by inducing the expression and release of damage associated molecular patterns (DAMPs). Thus, the specific immunity of the body will be activated (Korbelik et al., 2014).

The majority of studies have primarily focused on ICD, nanoparticles, drug delivery systems, and DC. ICD serves as the intellectual foundation of photodynamic therapy (PDT)-related immune responses. Moreover, an increasing number of studies have explored novel strategies to enhance antitumor immunity based on PDT, including the utilization of nanomaterial-based drug delivery systems, DC vaccines, and combination therapy with immune checkpoint inhibitors (ICIs).

Photodynamic therapy (PDT) has the ability to induce ICD and activate the antitumor immune response. Emerging evidence suggests that PDT enhances antitumor immunity, leading to the conversion of “cold” tumors into “hot” tumors (Falk-Mahapatra and Gollnick, 2020; Alzeibak et al., 2021). The ICD triggered by PDT results in the release of DAMPs that activate the innate immune system (Yang et al., 2017). These DAMPs, including heat shock proteins (HSPs), calreticulin (CRT), high mobility group box 1(HMGB1), and adenosine triphosphate (ATP), are recognized by pattern recognition receptors (PRRs) expressed on immune cells, subsequently promoting dendritic cell DC maturation. Mature DCs then activate adaptive immunity, stimulating naive T cells to differentiate into tumor-specific cytotoxic T cells and ultimately leading to tumor cell death (Tan et al., 2022). The IL10 gene promoter plays a critical role in the immune suppression induced by PDT (Gollnick et al., 1997). Following PDT, activator protein-1 (AP-1), a transcription factor, is activated in murine keratinocytes, regulating the IL10 gene promoter (Gollnick et al., 2001).

DC vaccines based on ICD have shown promising results in improving the activation and infiltration of tumor-specific T cells, while also reducing the presence of regulatory T cells (Tregs) within the tumor microenvironment (Garg et al., 2016). In several preclinical studies, the administration of DC that were cocultured with tumor cells treated with PDT (using hypericin or 5-ALA) resulted in improved survival outcomes in animals harboring specific tumors (Trempolec et al., 2020). These findings suggest that PDT-based DC vaccines have the potential to be translated into clinical applications for personalized tumor immunotherapy.

The main adverse effect of PDT is photosensitive reaction (Rkein and Ozog, 2014). For a few days after treatment, patients may experience local temporary reactive edema at the treatment site, which may also cause some discomfort, such as chest, back or abdominal pain, dyspnea in patients with bronchial cancer, dysphagia in patients with esophageal cancer, frequent urination and hematuria in patients with bladder cancer, and other possible side effects such as fever and constipation (Fitzmaurice and Eisen, 2016). All of these vary according to the lesion site and condition of the specific treatment, but they are generally not serious, their duration is short, and most can be alleviated by conventional management (Kwiatkowski et al., 2018). In general, the toxic side effects of PDT can be said to be very light, the skin’s photosensitive reaction can be completely prevented by avoiding light, and photodynamic therapy will not damage the function of the hematopoietic system and the immune system (Prażmo et al., 2016).

In recent years, immune checkpoint inhibitors (ICI) therapy has emerged as a promising approach for cancer immunotherapy. However, the effectiveness of ICI therapy heavily relies on the presence of infiltrated effector T cells within the tumor (Wu et al., 2022). Immune “cold” tumors are characterized by an immunosuppressive tumor microenvironment (TME) and a lack of infiltrating effector T cells, which significantly impacts the efficacy of ICI therapy (Galon and Bruni, 2019). Therefore, it has become crucial to modify the immune contexture of cold tumors and enhance weak antitumor immune responses. The combination of PDT with ICI therapy has shown synergistic effects and improved long-term prognosis (Galon and Bruni, 2019). PDT not only has a direct killing effect on tumors, but also can activate the body’s immune system. It is mainly carried out from two aspects: enhancing local immune response and activating adaptive immunity after PDT, and finally enhancing the killing effect of systemic immunity on tumor cells (Canti et al., 2002). The destruction of tumor tissue by PDT can cause local inflammatory response, contain and remove necrotic tissue, and restore normal tissue function and homeostasis. PDT causes the release of prostaglandins, leukotrienes and thrombolane, as well as the rapid upregulation of inflammatory cytokines such as IL-6, IL-1β and TNF-α and the activation of complement (Deng et al., 2023). After PDT, adaptive immunity can also be enhanced, resulting in the release of neutrophils, macrophages, NK cells, dendritic cells, *etc.*, destroying tumor cells while activating specific anti-tumor CD8^+^T cells, increasing the killing effect of local and systemic immune system on tumor cells (Kleinovink et al., 2017). Studies have suggested that PDT can improve the sensitivity of tumor to ICI, and PDT and CTLA-4 immune checkpoint inhibitors have synergistic anti-tumor effects (Cramer et al., 2020). Therefore, PDT combined with immunotherapy has a stronger killing effect on tumor cells. This combined approach not only eliminates the *in situ* tumor but also helps prevent tumor recurrence and metastasis (Cunzhi, 2022). Research on the combination of PDT with ICI therapy is rapidly expanding and the combination with PDT opens up another avenue for ICI therapy in the treatment of malignant tumors, especially in the treatment of metastatic tumors, and this treatment approach can achieve a synergistic effect on tumor treatment (Yu et al., 2023).

The diseases studied in the published literature predominantly focused on surface tissues, such as basal cell carcinoma, cutaneous squamous cell carcinoma, and intraepithelial neoplasia, primarily due to the limited penetration depth of near-infrared light (NIR) (Ng et al., 2018). Furthermore, the low tumor targeting capacity of photosensitizers (PS) imposes constraints on the effectiveness of PDT for treating cancer. Consequently, an increasing number of studies have directed their attention towards improving PS delivery for deep solid tumors through the use of multifunctional nanomaterial-based systems (Yu et al., 2022). In a recent study, a nanomaterial composed of photo-triggered gadofullerene has been designed to induce malignant tumor vascular disruption by enhancing the generation of reactive oxygen species (ROS). Moreover, this photo-triggered gadofullerene has been found to activate the immune response by facilitating DC maturation, promoting the differentiation of CD4^+^ and CD8^+^ T lymphocytes, and stimulating the release of proinflammatory factors (Guan et al., 2019). Additionally, to address the issue of hypoxia within the TME, novel nanoparticles incorporating catalase have been introduced to counteract the hypoxic conditions and ensure sufficient ROS generation from the targeted tissues.

## 5 Conclusion

In summary, this research highlights the importance of ICD, nanoparticles, drug delivery systems, and DC in enhancing antitumor immunity of PDT. Combining PDT with ICI therapy shows great potential for improving treatment outcomes, particularly in converting cold tumors into hot tumors. By understanding these key aspects, researchers can further explore personalized tumor immunotherapy strategies and pave the way for future breakthroughs in cancer treatment.

## Data Availability

The original contributions presented in the study are included in the article/Supplementary material, further inquiries can be directed to the corresponding authors.

## References

[B1] Agostinis P. Berg K. Cengel K. A. Foster T. H. Girotti A. W. Gollnick S. O. (2011). Photodynamic therapy of cancer: an update. CA Cancer J. Clin. 61 (4), 250–281. 10.3322/caac.20114 21617154 PMC3209659

[B2] Alzeibak R. Mishchenko T. A. Shilyagina N. Y. Balalaeva I. V. Vedunova M. V. Krysko D. V. (2021). Targeting immunogenic cancer cell death by photodynamic therapy: past, present and future. J. Immunother. Cancer 9 (1), e001926. 10.1136/jitc-2020-001926 33431631 PMC7802670

[B3] Canti G. De Simone A. Korbelik M. (2002). Photodynamic therapy and the immune system in experimental oncology. Photochem Photobiol. Sci. 1 (1), 79–80. 10.1039/b109007k 12659153

[B4] Cramer G. M. Moon E. K. Cengel K. A. Busch T. M. (2020). Photodynamic therapy and immune checkpoint blockade(†). Photochem Photobiol. 96 (5), 954–961. 10.1111/php.13300 32573787 PMC12435613

[B5] Cunzhi L. (2022). Photodynamic therapy combined with PD-1 inhibitor for bronchial tumors: one case report and literature review. J. Oncol. 2. 10.52768/2692-563x/1071

[B6] Deets K. A. Vance R. E. (2021). Inflammasomes and adaptive immune responses. Nat. Immunol. 22 (4), 412–422. 10.1038/s41590-021-00869-6 33603227

[B7] Deng B. Wang K. Zhang L. Qiu Z. Dong W. Wang W. (2023). Photodynamic therapy for inflammatory and cancerous diseases of the intestines: molecular mechanisms and prospects for application. Int. J. Biol. Sci. 19 (15), 4793–4810. 10.7150/ijbs.87492 37781521 PMC10539702

[B8] Donohoe C. Senge M. O. Arnaut L. G. Gomes-da-Silva L. C. (2019). Cell death in photodynamic therapy: from oxidative stress to anti-tumor immunity. Biochim. Biophys. Acta Rev. Cancer 1872 (2), 188308. 10.1016/j.bbcan.2019.07.003 31401103

[B9] Dumauthioz N. Labiano S. Romero P. (2018). Tumor resident memory T cells: new players in immune surveillance and therapy. Front. Immunol. 9, 2076. 10.3389/fimmu.2018.02076 30258445 PMC6143788

[B10] Falk-Mahapatra R. Gollnick S. O. (2020). Photodynamic therapy and immunity: an update. Photochem Photobiol. 96 (3), 550–559. 10.1111/php.13253 32128821 PMC7293553

[B11] Fitzmaurice S. Eisen D. B. (2016). Daylight photodynamic therapy: what is known and what is yet to be determined. Dermatol Surg. 42 (3), 286–295. 10.1097/DSS.0000000000000633 26918967

[B12] Galon J. Bruni D. (2019). Approaches to treat immune hot, altered and cold tumours with combination immunotherapies. Nat. Rev. Drug Discov. 18 (3), 197–218. 10.1038/s41573-018-0007-y 30610226

[B13] Garg A. D. Vandenberk L. Koks C. Verschuere T. Boon L. Van Gool S. W. (2016). Dendritic cell vaccines based on immunogenic cell death elicit danger signals and T cell-driven rejection of high-grade glioma. Sci. Transl. Med. 8 (328), 328ra27. 10.1126/scitranslmed.aae0105 26936504

[B14] Gollnick S. O. Lee B. Y. Vaughan L. Owczarczak B. Henderson B. W. (2001). Activation of the IL-10 gene promoter following photodynamic therapy of murine keratinocytes. Photochem. Photobiol. 73 (2), 170–177. 10.1562/0031-8655(2001)073<0170:aotigp>2.0.co;2 11272731 PMC2919222

[B15] Gollnick S. O. Liu X. Owczarczak B. Musser D. A. Henderson B. W. (1997). Altered expression of interleukin 6 and interleukin 10 as a result of photodynamic therapy *in vivo* . Cancer Res. 57 (18), 3904–3909. 9307269

[B16] Guan M. Zhou Y. Liu S. Chen D. Ge J. Deng R. (2019). Photo-triggered gadofullerene: enhanced cancer therapy by combining tumor vascular disruption and stimulation of anti-tumor immune responses. Biomaterials 213, 119218. 10.1016/j.biomaterials.2019.05.029 31136911

[B17] Kleinovink J. W. Fransen M. F. Löwik C. W. Ossendorp F. (2017). Photodynamic-immune checkpoint therapy eradicates local and distant tumors by CD8(+) T cells. Cancer Immunol. Res. 5 (10), 832–838. 10.1158/2326-6066.CIR-17-0055 28851692

[B18] Korbelik M. Banáth J. Sun J. Canals D. Hannun Y. A. Separovic D. (2014). Ceramide and sphingosine-1-phosphate act as photodynamic therapy-elicited damage-associated molecular patterns: cell surface exposure. Int. Immunopharmacol. 20 (2), 359–365. 10.1016/j.intimp.2014.03.016 24713544 PMC4043304

[B19] Kwiatkowski S. Knap B. Przystupski D. Saczko J. Kędzierska E. Knap-Czop K. (2018). Photodynamic therapy - mechanisms, photosensitizers and combinations. Biomed. Pharmacother. 106, 1098–1107. 10.1016/j.biopha.2018.07.049 30119176

[B20] Lu Y. Sun W. Du J. Fan J. Peng X. (2023). Immuno-photodynamic therapy (IPDT): organic photosensitizers and their application in cancer ablation. JACS Au 3 (3), 682–699. 10.1021/jacsau.2c00591 37006765 PMC10052235

[B21] Moghissi K. Dixon K. Thorpe J. A. Stringer M. Moore P. J. (2000). The role of photodynamic therapy (PDT) in inoperable oesophageal cancer. Eur. J. Cardiothorac. Surg. 17 (2), 95–100. 10.1016/s1010-7940(99)00350-4 10731642

[B22] Ng C. W. Li J. Pu K. (2018). Recent progresses in phototherapy-synergized cancer immunotherapy. Adv. Funct. Mater. 28 (46), 1804688. 10.1002/adfm.201804688

[B23] Nseyo U. O. (1989). “Immune response following photodynamic therapy for bladder cancer,” in Photodynamic therapy: mechanisms (SPIE). 10.1117/12.978005

[B24] Prażmo E. J. Kwaśny M. Łapiński M. Mielczarek A. (2016). Photodynamic therapy as a promising method used in the treatment of oral diseases. Adv. Clin. Exp. Med. 25 (4), 799–807. 10.17219/acem/32488 27629857

[B25] Pulendran B. Davis M. M. (2020). The science and medicine of human immunology. Science 369 (6511), eaay4014. 10.1126/science.aay4014 32973003 PMC7872131

[B26] Rkein A. M. Ozog D. M. (2014). Photodynamic therapy. Dermatol Clin. 32 (3), 415–425. x. 10.1016/j.det.2014.03.009 24891062

[B27] Small H. (1973). Co-citation in the scientific literature: a new measure of the relationship between two documents. J. Am. Soc. Inf. Sci. 24 (4), 265–269. 10.1002/asi.4630240406

[B28] Tan L. Shen X. He Z. Lu Y. (2022). The role of photodynamic therapy in triggering cell death and facilitating antitumor immunology. Front. Oncol. 12, 863107. 10.3389/fonc.2022.863107 35692783 PMC9184441

[B29] Trempolec N. Doix B. Degavre C. Brusa D. Bouzin C. Riant O. (2020). Photodynamic therapy-based dendritic cell vaccination suited to treat peritoneal mesothelioma. Cancers (Basel) 12 (3), 545. 10.3390/cancers12030545 32120810 PMC7139796

[B30] Wu Q. Chen Y. Li Q. Chen J. Mo J. Jin M. (2022). Time rules the efficacy of immune checkpoint inhibitors in photodynamic therapy. Adv. Sci. 9 (21), 2200999. 10.1002/advs.202200999 PMC9313507 35470595

[B31] Xu J. Xu L. Wang C. Yang R. Zhuang Q. Han X. (2017). Near-infrared-triggered photodynamic therapy with multitasking upconversion nanoparticles in combination with checkpoint blockade for immunotherapy of colorectal cancer. ACS Nano 11 (5), 4463–4474. 10.1021/acsnano.7b00715 28362496

[B32] Yang G. Xu L. Chao Y. Xu J. Sun X. Wu Y. (2017). Hollow MnO2 as a tumor-microenvironment-responsive biodegradable nano-platform for combination therapy favoring antitumor immune responses. Nat. Commun. 8 (1), 902. 10.1038/s41467-017-01050-0 29026068 PMC5638920

[B33] Yu X.-T. Sui S. Y. He Y. X. Yu C. H. Peng Q. (2022). Nanomaterials-based photosensitizers and delivery systems for photodynamic cancer therapy. Biomater. Adv. 135, 212725. 10.1016/j.bioadv.2022.212725 35929205

[B34] Yu Y. Yu R. Wang N. Bai Y. Shi Q. Maswikiti E. P. (2023). Photodynamic therapy in combination with immune checkpoint inhibitors plus chemotherapy for first-line treatment in advanced or metastatic gastric or gastroesophageal junction cancer: a phase 2-3 clinical trial protocol. Front. Pharmacol. 14, 1063775. 10.3389/fphar.2023.1063775 36778024 PMC9908746

[B35] Zou H. Wang F. Zhou J. J. Liu X. He Q. Wang C. (2020). Application of photodynamic therapy for liver malignancies. J. Gastrointest. Oncol. 11 (2), 431–442. 10.21037/jgo.2020.02.10 32399283 PMC7212095

